# Prediction of Prefecture-Level Subjective Well-Being in Japan by Using Google Trends and Socioeconomic Data: Machine Learning Model Development and Validation Study

**DOI:** 10.2196/88696

**Published:** 2026-03-20

**Authors:** Kenichi Kishi, Hisashi Hayashi, Shigeomi Koshimizu

**Affiliations:** 1 Advanced Institute of Industrial Technology Tokyo Japan

**Keywords:** infodemiology, infoveillance, subjective well-being, Google Trends, Japan, machine learning, stacked ensemble, forecasting, socioeconomic factors, digital trace

## Abstract

Incorporating prespecified Google Trends indicators into leakage-controlled stacked-ensemble models improved a 2025 holdout prediction of subjective well-being by using 2022-2025 data from Japan’s 47 prefectures, reducing the mean squared error from 0.0050 to 0.0045.

## Introduction

Subjective well-being (SWB) increasingly complements traditional economic indicators in evidence-based policymaking. Although Japan’s Digital Agency has published annual prefecture-level SWB aggregates (0-10 scale) since 2022 [1], the low reporting frequency limits real-time monitoring and timely policy intervention. Infodemiology involves the study of online information patterns, while infoveillance applies these patterns for health and social monitoring [2]. Notably, the Google Trends search activity can be leveraged as a demand-side infoveillance signal for evaluating prefecture-level SWB.

Google Trends signals have been linked to well-being and SWB nowcasting [3,4], but these data streams can exhibit high volatility at finer geographic scales [5]. Digital-trace SWB studies have also leveraged social media streams (eg, Twitter) [6]. In Japan, prefecture-level keyword queries are often limited by inadequate information [7]; we hypothesized that prespecified indicators using standardized categories and topic IDs can be implemented to improve reproducibility.

Herein, we extend prior digital-trace SWB nowcasting work by focusing on subnational (prefecture-level) prediction in Japan by using prespecified Google Trends category/topic identifiers to improve reproducibility and by evaluating performance under a strict temporal holdout year (2025). This study aims to evaluate whether prespecified Google Trends indicators provide incremental predictive value for prefecture-level evaluative SWB in Japan beyond socioeconomic and temporal predictors by using leakage-controlled stacked-ensemble modeling and strict 2025 holdout validation.

## Methods

This study uses publicly available, aggregated, nonidentifiable prefecture-level indicators; therefore, ethical review was not required.

We analyzed a prefecture-year panel (47 prefectures, 2022-2025) of published prefecture-year mean evaluative SWB (0-10) from the Digital Agency’s opt-in online survey [1] with leakage-controlled walk-forward stacked-ensemble modeling and a strict 2025 holdout (training 2022-2024, n=141 prefecture-years; holdout 2025, n=47).

Predictors were evaluated in three nested stages: stage 1 included socioeconomic indicators [8], stage 2 added temporal controls, and stage 3 incorporated Google Trends features. All variables are listed in Table S1 of Multimedia Appendix 1.

Model specification and validation reporting items aligned with TRIPOD+AI (Transparent Reporting of a Multivariable Prediction Model for Individual Prognosis Or Diagnosis with Artificial Intelligence) are summarized in Table S2 of Multimedia Appendix 1.

We prespecified 25 Google Trends category/topic series by mapping OECD (Organization for Economic Co-operation and Development) well-being domains [9] to Google Trends categories and topic IDs and retrieving indices via PyTrends (Table S3 in Multimedia Appendix 1).

Trends were summarized using principal component analysis (>90% variance), and the outcomes and descriptive comparability summaries are presented in Tables S4 and S5 of Multimedia Appendix 1.

For interpretability, we ranked raw series by association with the first Trends component (principal component 1) and refit ElasticNet and extreme gradient boosting (XGBoost) by using the top 8 series.

For validation, walk-forward stacking (2022→2023, 2022-2023→2024) was employed with within-window preprocessing and tuning to avoid leakage; we report adjusted *R*^2^ and mean squared error (MSE) with 95% bootstrap CIs for 2025 (B=4000), following TRIPOD+AI guidance [10].

Algorithm S1 (Multimedia Appendix 2) and the analysis repository (Multimedia Appendix 3) support the replication.

## Results

**Figure 1:**
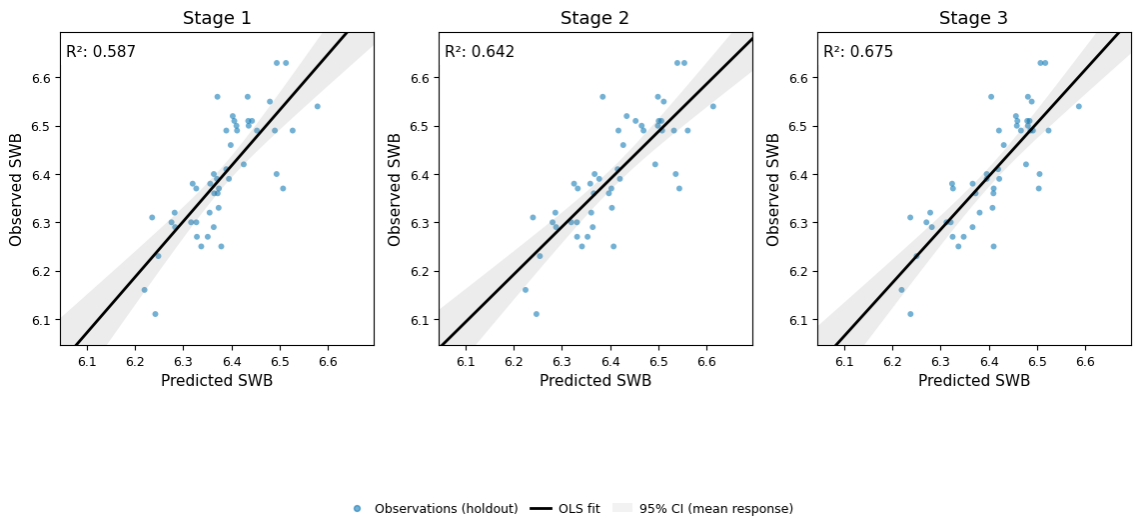
Observed vs predicted 2025 prefectural subjective well-being (SWB) at stages 1-3. OLS: ordinary least squares.

On the 2025 holdout, the adjusted *R*^2^ increased from 0.587 (stage 1) to 0.642 (stage 2) and 0.675 (stage 3), and the MSE decreased from 0.0050 to 0.0045 (Figure 1). The bootstrap 95% CIs for the adjusted *R*^2^ overlapped.

Sensitivity refits using the 8 principal component 1–aligned raw series did not improve performance compared with the principal component analysis–based Trends specification (Table S6 in Multimedia Appendix 1).

**Figure 2:**
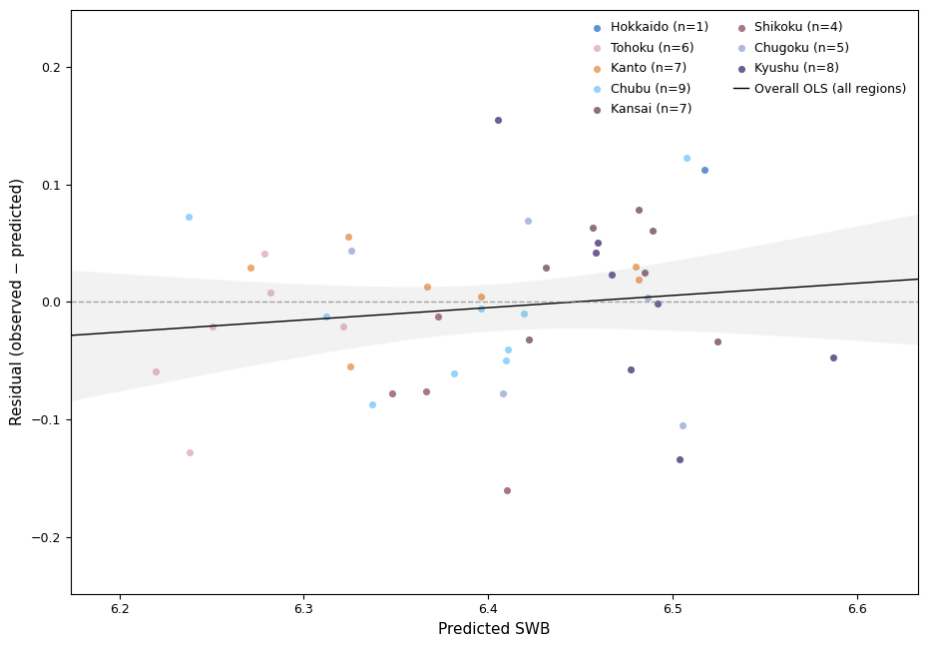
Residuals vs predicted 2025 subjective well-being (SWB) for the final model, by region. OLS: ordinary least squares.

Calibration yielded a slope of 1.10 (95% CI 0.88-1.33) and an intercept of −0.67 (95% CI −2.12 to 0.78) (Figure 2). Furthermore, the prefecture-level MSE ranged from 0.000003 to 0.0257 (median 0.0022, n=47), with no strong regional bias (Figure S1 in Multimedia Appendix 4).

## Discussion

Adding prespecified Google Trends components to socioeconomic and temporal predictors modestly improved the strict 2025 holdout prediction (adjusted *R*^2^=0.642-0.675, MSE=0.0050-0.0045). This result demonstrates the incremental value of search-derived indicators as an exploratory signal for prefecture-level SWB infoveillance [2].

Compared with national SWB nowcasting using Google Trends [4], subnational prediction with annual outcomes may be limited by lower signal-to-noise ratios. Standardized categories and topic IDs were exploited to improve reproducibility and reduce sparsity relative to keyword queries [7]. The dominant component reflected a broad lifestyle/mobility/consumption factor, and the ranked categories are provided in Table S6 of Multimedia Appendix 1 for hypothesis-driven follow-up. Note that these outputs should be treated as a cautious predeployment screening signal rather than a ranking or policy decision tool.

Limitations include the short panel (4 years), the ecological outcome, and reliance on a nonprobability opt-in survey with limited publicly disclosed quality-control details and uncertain year-to-year comparability (Tables S4 and S5 in Multimedia Appendix 1). Because only aggregated prefecture-year means are publicly available, we could not independently assess instrument reliability or test measurement invariance across years. Google Trends indices are normalized and may be sampled, and unmeasured time-varying factors may confound associations. Accordingly, findings should not be interpreted as causal.

Under leakage-controlled temporal validation, Google Trends signals added modest predictive value for prefecture-level evaluative SWB in Japan. Future work should test higher-frequency outcomes, lagged designs, and external validation to clarify when such signals are the most informative.

## Data Availability

All data are publicly available (Digital Agency [1], e-Stat [8], Google Trends); the code and materials are described in Multimedia Appendix 4.

## Appendix

Multimedia Appendix 1 Detailed variable definitions, TRIPOD-AI reporting checklist, Google Trends identifiers, subjective well-being outcome transparency documentation, year-to-year comparability summaries, and sensitivity analyses.

Multimedia Appendix 2 Algorithm S1 (walk-forward stacking pseudocode): step-by-step pseudocode for the leakage-controlled walk‑forward training and evaluation pipeline, including preprocessing, feature engineering, and stacked‑ensemble integration for predicting prefectural subjective well-being.

Multimedia Appendix 3 Full analysis repository (code and project root): this archive contains the full project root, including source code, configuration files, and the analysis pipeline used to reproduce the walk‑forward stacked‑ensemble models for prefectural subjective well-being prediction.

Multimedia Appendix 4 Prefecture-level mean squared error for 2025 holdout.

Multimedia Appendix 5 TRIPOD-AI–oriented reporting checklist. This checklist maps key aspects of the study (data sources, predictors, outcomes, model evaluation, robustness, fairness, reproducibility, and intended use) to TRIPOD‑AI items to support transparent reporting of the prediction model.

## Abbreviations

MSE: mean squared error
OECD: Organization for Economic Co-operation and Development
SWB: subjective well-being
TRIPOD+AI: Transparent Reporting of a Multivariable Prediction Model for Individual Prognosis Or Diagnosis with Artificial Intelligence
XGBoost: extreme gradient boosting

## References

[1] Utilizing the liveable well-being city indicator: towards the realization of the digital garden city-state vision. Digital Agency, Government of Japan.

[2] Eysenbach G (2009). Infodemiology and infoveillance: framework for an emerging set of public health informatics methods to analyze search, communication and publication behavior on the Internet. J Med Internet Res.

[3] Algan Y, Murtin F, Beasley E, Higa K, Senik C (2019). Well-being through the lens of the internet. PLoS One.

[4] Murtin F, Salomon-Ermel M (2024). Nowcasting subjective well-being with Google Trends: a meta-learning approach. OECD Papers on Well-being and Inequalities.

[5] Rovetta A (2024). Google trends in infodemiology: methodological steps to avoid irreproducible results and invalid conclusions. Int J Med Inform.

[6] Carpi T, Hino A, Iacus SM, Porro G (2023). The impact of COVID-19 on subjective well-being: evidence from Twitter data. J Data Sci.

[7] Yang MS, Taira K (2024). Predicting prefecture-level well-being indicators in Japan using search volumes in internet search engines: infodemiology study. J Med Internet Res.

[8] Statistics Bureau of Japan e-Stat: Portal Site of Official Statistics of Japan.

[9] (2013). OECD guidelines on measuring subjective well-being. Organisation for Economic Co-operation and Development.

[10] Collins GS, Moons KGM, Dhiman P, Riley RD, Beam AL, Van Calster B, TRIPOD+AI Group (2024). TRIPOD+AI statement: updated guidance for reporting clinical prediction models that use regression or machine learning methods. BMJ.

