# Supplementary material for: Prediction of Prefecture-Level Subjective Well-Being in Japan by Using Google Trends and Socioeconomic Data: Machine Learning Model Development and Validation Study
Source: JMIR Form Res. 2026 Mar 20;10:e88696. doi: 10.2196/88696 (PMC13049395; doi:10.2196/88696)
Supplement: Multimedia Appendix 1 [file formative_v10i1e88696_app1.pdf]

Table S1.Full variable list and feature blocks used in Stages 1–3.

Feature block	Variables (code in analysis)	Description	Used in
PREFECTURE_i (prefecture fixed effects)	pref_emb_1–pref_emb_6	Low-dimensional learned embeddings representing prefecture fixed effects (implementation of PREFECTURE_i used as inputs for base learners).	Stages 1–3 (base)
PREFECTURE_i (prefecture fixed effects)	pref_Aichi, …, pref_Okinawa (46 dummies; one reference omitted)	Explicit prefecture dummy indicators used only in the meta-learner to estimate prefecture-specific intercept adjustments consistent with PREFECTURE_i.	Stages 1–3<br>Meta-learner only
PREFECTURE_i (region)	region_Hokkaido, region_Tohoku, region_Kanto, region_Chubu, region_Kansai, region_Chugoku, region_Shikoku, region_Kyushu (drop-one coding)	Macro-region dummy variables (aligned to Japanese regional classification).	Stages 1–3
SOCIO_{i,t} (socioeconomic indicators)	cpi_value	Prefecture-level consumer price index (CPI).	Stages 1–3
SOCIO_{i,t} (socioeconomic indicators)	gpp_2021	Gross prefectural product (GPP), 2021.	Stages 1–3
SOCIO_{i,t} (socioeconomic indicators)	income_per_capita_kjpy_2021	Income per capita (thousand JPY), 2021.	Stages 1–3
SOCIO_{i,t} (socioeconomic indicators)	labor_force_q1	Labor force size (first-quarter estimate).	Stages 1–3
SOCIO_{i,t} (socioeconomic indicators)	labor_force_rate	Labor force participation rate.	Stages 1–3
SOCIO_{i,t} (socioeconomic indicators)	labor_participation_rate_1q	Labor force participation rate (first quarter).	Stages 1–3
TIME_t (temporal controls)	time_idx_c	Centered time index (years; baseline 'clock').	Stages 2–3
TIME_t (temporal controls)	yr_sin_p2, yr_cos_p2; yr_sin_p4, yr_cos_p4	Yearly Fourier sine/cosine terms with periods 2 and 4 years.	Stages 2–3
TIME_t (temporal controls)	ets_pred	One-step-ahead Simple Exponential Smoothing (ETS) forecast of SWB.	Stages 2–3
TIME_t (temporal controls)	nikkei225	Nikkei 225 index series (non–prefecture-specific; varies by year and is shared across all prefectures) as a temporal/macro control	Stages 2–3
TIME_t (SWB history)	pref_trend_slope, pref_volatility	Per-prefecture linear slope and standard deviation of SWB over 2022–2024.	Stage 3
TRENDS_{i,t} (Google Trends)	g_pca1–g_pca16	Principal components of prefecture-level Google Trends SVI; number retained chosen to exceed 90% cumulative explained variance (capped at 100).	Stage 3
TRENDS_{i,t} (Google Trends – shift stats)	g_pca_shift_mean_abs, g_pca_shift_l2, g_pca_shift_max_abs	Shift statistics computed by standardizing 2025 apply-set g_pca against training means/SDs.	Stage 3
TRENDS×SOCIO interactions	ix__g_pca1–5__x__gpp_2021;<br>ix__g_pca1–5__x__labor_force_q1;<br>ix__g_pca6–16__x__labor_force_q1;<br>ix__g_pca_shift_*__x__labor_force_q1	Unsupervised top-k pairwise products between Trends PCs (and their shift stats) and selected SOCIO variables (see Methods).	Stage 3

Table S2.TRIPOD+AI Checklist

Section	Item	Description
A. Study Design Transparency	1. Data source & timeframe	Publicly released prefecture-level aggregated panel (47 prefectures × 2022–2025). Training: 2022–2024 (N=141 prefecture-years); held-out evaluation: 2025 (N=47). Outcome: prefecture-level mean SWB (0–10) from the Digital Agency's Well-Being Dashboard (measurement/aggregation and provenance: Table S4). Predictors: e-Stat socioeconomic indicators, temporal controls, and Google Trends indices retrieved via PyTrends and summarized with PCA (>90% variance). Google Trends category/topic definitions (cat IDs, MIDs, and sparsity fallback) are documented in Table S3; the full variable list appears in Table S1 (Multimedia Appendix).
	2. Participants/units & sampling	Unit of analysis: prefecture-year (all 47 prefectures; no sampling at the analytical unit). The underlying SWB survey uses an opt-in online panel with quota recruitment; conventional response rates are not defined. Disclosure items related to survey mode/quotas and outcome construction are summarized in Table S4-S5.
	3. Predictors	Predictors were grouped into blocks and evaluated in nested feature sets (Stages 1→2→3). Stage 1: fixed effects + socioeconomic indicators (e-Stat). Stage 2: Stage 1 + temporal controls (centered time index, exponential-smoothing forecast, annual Fourier terms). Stage 3: Stage 2 + Google Trends components. Google Trends indicators were retrieved via PyTrends and reduced via PCA retaining components explaining ≥90% of variance; limited interactions with selected socioeconomic variables were used. Category/topic selection and IDs/MIDs (incl. fallback to category-level series when sparse) are documented in Table S3.
	4. Outcome	Outcome was prefecture-level mean subjective well-being (SWB; 0–10) from the Digital Agency's annual survey, analyzed as publicly released prefecture-level aggregates. Per official clarification, prefecture means are (i) simple (unweighted) averages of individual records, (ii) computed directly by aggregating individual responses to prefecture (not by re-averaging municipality means), and (iii) exclude "no answer"/missing responses. See Table S4 for fieldwork dates, survey mode/quotas, IP/terms notes, and the data retrieval date used for this study.
B. Model Evaluation & Reproducibility	5. Models (rationale)	Base learners (7) were chosen to span distinct model families and reduce model-specification risk: regularized linear models (Ridge, Elastic Net), a kernel-based nonlinear model (SVR), and tree/boosting ensembles (Extra Trees, XGBoost, LightGBM shallow and deep). Stacking combines complementary inductive biases; the meta-learner was Elastic Net for stability in a small-N panel. Meta-input filtering retained a capped subset of weakly correlated base predictions to preserve diversity and avoid redundancy. Implementation details, software versions, and configs are provided in Multimedia Appendix (project-root.zip; requirements.txt).
	6. Data splitting & preprocessing	Leakage-controlled walk-forward design. Fold 1 trained on 2022 and validated on 2023; Fold 2 trained on 2022–2023 and validated on 2024. Out-of-fold base predictions were concatenated to train the second-layer Elastic Net meta-learner. Year 2025 was reserved as a strict held-out evaluation set. Preprocessing (standardization and median imputation) was fit within each training fold and applied to the corresponding validation fold to avoid leakage. Low-variance and multicollinearity diagnostics (eg, variance checks and VIF/correlation) were monitored, but predictors were not excluded based on these diagnostics. Hyperparameters were tuned using only the training years within each outer fold (nested).
	7. Performance metrics	Performance was summarized using adjusted R^2 and mean squared error (MSE). Holdout uncertainty was summarized with 95% bootstrap confidence intervals (B=4000) for adjusted R^2 and MSE.
	8. Calibration	Calibration on the 2025 holdout year was assessed using recalibration slope/intercept and residual-vs-predicted plots; see main text Figure 2 (region-annotated residual plot).
	9. Incremental value	Incremental value of predictor blocks was quantified using nested feature sets (Stage 1: fixed effects + socioeconomic; Stage 2: + temporal controls; Stage 3: + Google Trends) and summarized as block-wise adjusted ΔR^2 ("information gain").
	10. Robustness	Robustness checks included (i) consistent evaluation across nested feature sets (Stages 1→2→3), (ii) stability diagnostics for Google Trends PCA loadings across expanding training windows, and (iii) sensitivity to base-model redundancy via correlation-based meta-input filtering (capped).
	11. Fairness/heterogeneity	Heterogeneity was assessed by inspecting region-level residual patterns in main text Figure 2. Prefecture-level MSEs with 95% bootstrap CIs (B=4000) for the 2025 holdout are provided as Supplementary Figure 1(Prefecture-level MSE for 2025 holdout) in Multimedia Appendix 3. Individual-level subgroup fairness cannot be assessed because the outcome is available only as prefecture-level aggregates.
	12. Reproducibility	The full analysis repository is provided as Multimedia Appendix (project-root.zip), including the complete project root (source code, configuration files, and requirements.txt) and the end-to-end pipeline (run_main.ipynb). The exact Google Trends category/topic definitions used in the study (category IDs and topic MIDs, including sparsity-fallback mappings) are provided as a metadata template (terms_template.csv). Raw Google Trends series and the Nikkei 225 series are not redistributed; researchers can obtain equivalent data from the original sources and populate the local SQLite database expected by the pipeline (see README).
	13. Limitations & intended use	Limitations include ecological inference risk, reliance on a non-probability online panel for the outcome (no conventional response rate; no official representativeness statement), and possible year-to-year survey/design differences (measurement stability across years is assumed for model training, but measurement invariance and reliability cannot be independently assessed from publicly released aggregates alone). Google Trends indices are normalized and may be sampled. Intended use is academic prediction/forecast evaluation rather than league-table ranking or policy decisions without additional validation, consistent with SCI-Japan/Digital Agency guidance on off-purpose use.

Table S3. Google Trends series selection, identifiers (category IDs/topic IDs), and a priori mapping to OECD well-being dimensions.

Category (Google Trends)	Category ID (cat_filter)	OECD well-being dimension(s) (a priori)	Retrieval mode	# topic series	Seed concepts (English; a priori)	Seed concepts (Japanese; documentation only)	Topic IDs (MID; semicolon-separated)	Category inclusion rationale (OECD-aligned)	Notes / fallback
Arts & Entertainment	3	Work-life balance (leisure), Community (cultural participation)	topic-level	5	film; television; music; concert; streaming media	映画; テレビ; 音楽; コンサート; ストリーミング	/m/02vxn; /m/07c52; /g/11cp7ksmnn; /m/01jddz; /m/0c6wml	Included a priori as a leisure/cultural participation proxy; entertainment-related searches may reflect mood-related activities and time use.	—
Internet & Telecommunications	13	Social connections, Education & skills (information access), Civic engagement	topic-level	5	social media; messaging; mobile data; Wi-Fi; cloud computing	SNS; メッセージング; モバイルデータ; Wi-Fi; クラウド	/g/11j8qrlk97; /m/02nf_; /g/11v_92j2mr; /m/0h4d9; /m/02y_9m3	Included a priori to capture the digital information environment and connectivity (social media, messaging, Wi-Fi), which shape social interaction and exposure to information relevant to well-being.	—
Online Communities	299	Social connections (community)	category-level	0	online community; internet forum; message board; online discussion; chat room	オンラインコミュニティ; インターネット掲示板; メッセージボード; オンライン議論; チャットルーム		Included a priori to capture online social interaction and community engagement; used as a category-level proxy where topic-level series were sparse or missing at prefecture resolution.	Used category-level series (cat=299) because topic-level series were sparse/missing at prefecture resolution.
Games	8	Work-life balance (leisure)	topic-level	5	game console; mobile game; esports; role-playing game; puzzle game	ゲーム機; モバイルゲーム; eスポーツ; RPG; パズル	/m/07_tb; /m/04951x; /m/02qh79; /m/0403l3g; /m/07gl9	Included a priori as a leisure-domain proxy; gaming-related searches may reflect recreational activities and coping behavior relevant to mood and time use.	—
Computers & Electronics	5	Education & skills (digital skills), Jobs & earnings (digital economy)	topic-level	5	laptop; smartphone; semiconductor; operating system; battery	ノートPC; スマートフォン; 半導体; OS; バッテリー	/m/01c648; /m/050k8; /m/08mh3kd; /m/05khh; /m/01c0z	Included a priori to capture technology adoption and digital access (devices, operating systems), which may relate to work, education, and daily convenience.	—
Shopping	18	Income & wealth (cost of living/consumption), Material conditions	topic-level	5	sale; coupon; online shopping; returns; price increase	セール; クーポン; ネット通販; 返品; 物価高	/g/113lz97yx; /m/02jfdl; /m/047m52; /m/044g_k; /g/11nxv3q91v	Included a priori as a proxy for consumption and price pressure (e.g., sales, returns, price increases), which relates to material conditions and daily stress.	—
Sports	20	Health, Work-life balance (leisure/physical activity)	topic-level	5	soccer; baseball; marathon; Olympic Games; fitness	サッカー; 野球; マラソン; オリンピック; フィットネス	/m/036bzt; /g/11c6t_1mq9; /m/0d38m; /m/05nd_; /g/11c6vfcdr1	Included a priori because physical activity and sports participation relate to health and positive affect; sports-related searches may proxy engagement in active lifestyles.	—
News	16	Civic engagement, Safety (shocks/disasters), Material conditions (macro news)	topic-level	5	inflation; economic outlook; infectious disease; natural disaster; election	物価; 景気; 感染症; 災害; 選挙	/m/09jx2; /g/11m59lwv6l; /m/03tp4; /m/0g2k1; /m/02l3h	Included a priori to capture attention to societal shocks and macro conditions (economic outlook, outbreaks, disasters), which can influence well-being through uncertainty and risk perception.	—
Business & Industry	12	Jobs & earnings, Income & wealth (macro economy)	topic-level	5	small business; subsidy; supply chain; inventory; price increase	中小企業; 補助金; サプライチェーン; 在庫; 値上げ	/g/11j317s7xg; /m/012qhn; /m/019h6j; /m/018y_6; /g/11nxv3q91v	Included a priori to capture macroeconomic and business-cycle signals (subsidies, supply chains, price hikes) relevant to employment and economic security.	—
Food & Drink	71	Health, Work-life balance (daily life)	topic-level	5	recipe; eating out; diet; cafe; allergy	レシピ; 外食; ダイエット; カフェ; アレルギー	/m/0p57p; /g/11gchzp7kx; /g/1213h4d9; /g/11ty189hzk; /g/11j8qr8r2t	Included a priori because diet-related behavior and food access relate to health and daily satisfaction; food-related searches may proxy routines and preferences.	—
Pets & Animals	66	Social connections, Health (mental well-being)	category-level	0	dog; cat; pet insurance; veterinarian; pet adoption	犬; 猫; ペット保険; 動物病院; 里親		Included a priori because pet ownership and animal-related activities can contribute to companionship and affective well-being; used as a broad proxy where topic-level data were sparse.	Used category-level series (cat=66) because topic-level series were sparse/missing at prefecture resolution.
Science	174	Education & skills, Environmental quality, Health (public health)	category-level	0	climate change; artificial intelligence; epidemiology; psychology; public health	気候変動; 人工知能; 疫学; 心理学; 公衆衛生		Included a priori to capture public attention to science/technology and public-health/environment topics that may shape expectations and perceived risks relevant to well-being.	Used category-level series (cat=174) because topic-level series were sparse/missing at prefecture resolution.
Finance	7	Income & wealth (financial security), Material conditions	topic-level	5	inflation; interest rate; exchange rate; household budget; mortgage	インフレ; 金利; 為替; 家計; 住宅ローン	/m/09jx2; /m/04n7dpf; /m/018m33; /g/11m5gpxc66; /m/0273t5w	Included a priori because financial security and cost-of-living pressures are strongly associated with subjective well-being; finance-related searches may proxy economic uncertainty and household constraints.	—
Health	45	Health (physical and mental)	topic-level	5	depression; anxiety disorder; sleep disorder; exercise; vaccination	うつ病; 不安障害; 睡眠障害; 運動; 予防接種	/m/01g2q; /m/0ftsr; /m/0cnmb; /m/05bvcl; /g/121j1nlf	Included a priori because physical and mental health are foundational determinants of subjective well-being; health-related search interest may proxy population health concerns.	—
Jobs & Education	958	Jobs & earnings, Education & skills	topic-level	5	unemployment; job openings; career change; salary; scholarship	失業; 求人; 転職; 給与; 奨学金	/m/07s_c; /g/11yg29r3pr; /g/11nnz9psh6; /m/02w88k; /m/018m77	Included a priori because employment and human capital are core determinants of material conditions and life satisfaction; related searches may proxy labor-market stress and opportunity.	—
Reference Materials	533	Safety (weather), Work-life balance (mobility/time use)	topic-level	5	weather; map; time; train schedule; translation	天気; 地図; 時刻; 路線; 翻訳	/m/0866r; /m/04_tb; /g/1yw7410rz; /g/1235w5lb; /m/07h26	Included a priori to capture routine information needs (weather, maps, transit schedules) that relate to daily functioning, mobility, and stress.	—
Automotive	47	Safety (transport), Work-life balance (mobility)	topic-level	5	used car; vehicle inspection; car insurance; electric vehicle; fuel economy	中古車; 車検; 保険; EV; 燃費	/m/0d4yhw; /g/11q3q4kfw6; /g/11fsrbwv27; /m/03bxwvx; /m/0410vz6	Included a priori to capture mobility and transportation-related costs/safety (vehicle inspection, insurance), which can influence daily burden and access.	—
Hobbies & Leisure	65	Work-life balance (leisure)	topic-level	5	travel planning; photography; camping; reading; movie watching	旅行計画; 写真; キャンプ; 読書; 映画鑑賞	/g/11ltj9yf_v; /m/068jd; /m/01h6d4; /g/11sdpt8dwj; /g/11vbq2tdxz	Included a priori to capture leisure planning and hobbies that contribute to work–life balance and positive affect.	—
Home & Garden	11	Housing (home conditions)	topic-level	5	housework; cleaning; gardening; home renovation; home appliances	家事; 掃除; ガーデニング; リフォーム; 家電	/g/11lkx5qz_1; /g/11fn051cn2; /m/034v_; /g/11j8qrg33h; /g/11fsrbwfs0	Included a priori to proxy home conditions and household routines (cleaning, renovation), which can affect comfort and stress in daily life.	—
Books & Literature	22	Education & skills, Work-life balance (leisure)	category-level	0	novel; manga; reading; library; book review	小説; 漫画; 読書; 図書館; 書評		Included a priori as a proxy for leisure/learning activities; used as a broad proxy where topic-level data were sparse.	Used category-level series (cat=22) because topic-level series were sparse/missing at prefecture resolution.
People & Society	14	Social connections (family/community)	topic-level	5	marriage; divorce; parenting; elder care; loneliness	結婚; 離婚; 子育て; 介護; 孤独	/m/04ztj; /g/11sr9_s2qj; /m/0fglh; /m/0czbt60; /m/05c7vv	Included a priori because social connections, family relations, and caregiving demands are key determinants of well-being; related searches may proxy social support or strain.	—
Beauty & Fitness	44	Health (lifestyle), Work-life balance	topic-level	5	diet; strength training; yoga; skin care; hair salon	ダイエット; 筋トレ; ヨガ; スキンケア; 美容院	/g/1213h4d9; /m/04lbbc; /g/11vm5dv0d; /g/122_gf1z; /g/11j317ps40	Included a priori because lifestyle and self-care behaviors relate to health and self-perception; related searches may proxy engagement in health-promoting routines.	—
Real Estate	29	Housing (affordability/stability), Material conditions	topic-level	4	rental; home purchase; rent; mortgage; property tax	賃貸; 住宅購入; 家賃; 住宅ローン; 固定資産税	/g/11q4c9js4r; /g/11j2fvgqzh; /m/0273t5w; /m/020shm	Included a priori because housing affordability and stability influence well-being; real-estate searches may proxy housing-market pressures and household planning.	—
Law & Government	19	Civic engagement & governance (institutions/policy)	topic-level	5	tax; tax return; pension; election; My Number	税金; 確定申告; 年金; 選挙; マイナンバー	/m/07g82; /g/11rtq707pn; /m/0by51; /m/02l3h; /g/11vj41kh5_	Included a priori because institutions and policy salience (e.g., taxation, pensions, elections) relate to civic engagement and perceived security; related searches may proxy policy-related concerns.	—
Travel	67	Work-life balance (leisure/travel), Social connections	topic-level	5	travel; hotel; airfare; tourist attractions; passport	旅行; ホテル; 航空券; 観光地; パスポート	/m/014dsx; /g/11g6qjncfz; /g/11m9zlvzkt; /m/0117n44z; /m/05sv1	Included a priori as a proxy for leisure mobility and vacation planning, which relate to work–life balance and social activity.	—

Table S4. Reporting and transparency checklist for the SWB outcome (prefecture-level "average happiness score," 0–10).

No.	Domain	Item (Yes/No question)	Disclosure (Digital Agency SWB / This study)
1	Measurement	[OECD SWB] Is evaluative SWB measured on an 11-point numeric scale (0–10), as generally recommended?	[Digital Agency SWB] The 'Happiness' item uses an 11-point 0–10 numeric scale.<br>[This study (outcome use)] Outcome = the published prefecture-year mean of this 0–10 'Happiness' item (no rescaling).
2	Measurement	[OECD SWB] Are scale anchors (endpoint labels) clearly defined?	[Digital Agency SWB] Endpoints are explicitly labeled: 0 = "Very unhappy"; 10 = "Very happy".<br>[This study (outcome use)] We use the anchors exactly as defined (see the exact item wording in No. 15).
3	Sampling & recruitment	[AAPOR TI 5a] Do we clearly state whether the survey sample is probability-based or nonprobability?	[Digital Agency survey] Data are collected via an opt-in online panel with quota targets; selection probabilities are not known ⇒ nonprobability sample.<br>[This study (outcome use)] We treat the outcome as derived from a nonprobability online survey and do not make probability-sample inference claims.
4	Sampling & recruitment	[AAPOR TI 5d] Do we disclose the use of quotas?	[Digital Agency survey] Quota targets are used for recruitment/collection.<br>[This study (outcome use)] Because quota/opt-in recruitment can induce selection and coverage bias, prefecture means are interpreted as descriptive indicators (not guaranteed unbiased population means).
5	Fieldwork period	[AAPOR TI 7] Are fieldwork dates disclosed (at least for each survey year used in the analysis)?	[Digital Agency survey (national SWB survey)] Fieldwork periods by year:<br>• 2022: ≈ June 2022 (month-level only; start/end dates not published)<br>• 2023: May 11–23, 2023<br>• 2024: May 14–20, 2024<br>• 2025: May 14–20, 2025<br>Note: 2022 has limited date precision (month-level only).
6	Sample size	[AAPOR TI 8a] Is the total sample size disclosed, including valid N by year (2022–2025)?	[Digital Agency survey] Valid responses by year (as publicly reported):<br>• 2022: ≈34,000<br>• 2023: 85,236<br>• 2024: 101,498<br>• 2025: 86,420<br>[This study (outcome use)] Analytic unit is prefecture-year means (47 prefectures × 4 years = 188 observations).
7	Sample size	[AAPOR TI 8a] Is the total sample size disclosed?	[Digital Agency survey] Total individual-level N across 2022–2025 is ≈307,000 (sum of public year-by-year valid counts; 2022 is approximate).<br>[This study (outcome use)] The analysis uses aggregated prefecture-year means; individual microdata are not used.
8	Uncertainty reporting	[AAPOR TI 8c] Do we avoid reporting a "margin of error (MOE)" for nonprobability samples (unless model-based)?	[Digital Agency survey] As a nonprobability online survey, a probability-sample MOE is not applicable.<br>[This study (outcome use)] We do not report probability-sample MOE. Any uncertainty we show is explicitly model-based (e.g., bootstrap / predictive error), not labeled as MOE.
9	Weighting	[AAPOR TI 9] If weights are used, are they described?	[Digital Agency survey] Prefecture-level 'Happiness' mean is a simple average of individual records; no weighting is applied.<br>[This study (outcome use)] We use the published prefecture means as-is (no additional weighting).
10	Missing data	[AAPOR TI 10] Is missing-data handling disclosed?	[Digital Agency survey] Prefecture means exclude missing/blank values for the SWB item.<br>[This study (outcome use)] Outcome definition follows the same rule: mean of non-missing SWB responses within prefecture-year.
11	Data quality / QC	[AAPOR TI 10] Are vendor quality-control procedures (duplicates/fraud/straightlining/response time, etc.) described?	[Digital Agency survey] The survey company conducts quality control (e.g., excluding fraudulent responses) to the extent possible; detailed rules (duplicates/fraud criteria, etc.) are not publicly disclosed.<br>[This study (outcome use)] Because QC details cannot be externally verified, residual risk of low-quality responses is treated as an explicit limitation.
12	Response metrics	[Response-rate concept] Is response rate reported or properly contextualized?	[Digital Agency survey] With opt-in panels and quota recruitment, a probability-sample response rate (e.g., AAPOR RR) is not defined.<br>[This study (outcome use)] No response rate is reported; selection/coverage bias is treated as a core limitation.
13	Missing data	[Item nonresponse] Is item nonresponse addressed?	[Digital Agency survey] Most items are mandatory; therefore item nonresponse does not occur for the SWB items used here (an exception is the 'town happiness' item, not used as this study's outcome).<br>[This study (outcome use)] The outcome uses the published prefecture mean; item-level nonresponse rates cannot be reconstructed from aggregated data.
14	Instrument	[AAPOR TI 3] Are the exact question wording and response options available?	[Digital Agency SWB] Item wording (Happiness): "Currently, how happy are you? If 10 is 'Very happy' and 0 is 'Very unhappy', what score would you give? Please answer with one number." Response: one integer from 0 to 10.<br>Source: Digital Agency, "Regional Well-Being Indicator—Survey Questionnaire (Standard model 50 items)" (Excel; updated 8 Jun 2023).<br>[This study (outcome use)] Outcome corresponds to the prefecture-level mean of this item as published on the dashboard.
15	Limitations	[AAPOR TI 11] Do we include a general limitations statement about unmeasured error?	[Digital Agency SWB] As a nonprobability online survey, unmeasured errors (self-selection, coverage, mode effects) may remain.<br>[This study (outcome use)] Prefecture means may be systematically biased relative to population means; results are interpreted as descriptive associations rather than unbiased population estimates.
16	Measurement	[OECD SWB] Is the construct clearly defined (evaluative vs affect vs eudaimonia, etc.)?	[Digital Agency SWB] Single-item 0–10 evaluative SWB ("Happiness").<br>[This study (outcome use)] The outcome reflects evaluative SWB; it is not a direct measure of affective SWB (momentary emotions) or eudaimonia (meaning/purpose).
17	Interpretation	[Use limitation] Do we explicitly discourage league-table ranking or causal policy claims from cross-sectional differences?	[SCI-Japan/Digital Agency guidance] The indicator is intended for use toward the Digital Garden City Nation initiative; league-table rankings and other off-purpose uses are discouraged.<br>[This study (outcome use)] We do not present league tables and do not make causal policy claims from cross-sectional prefecture differences; comparisons are descriptive.
18	Provenance & versioning	[Versioning/corrections] Do we document data version, retrieval date, and known corrections/changes?	[Digital Agency/Sci-Japan] Dashboard data and calculation methods may be updated/changed, and publication may be suspended.<br>[This study (outcome use)] Data snapshot used here was retrieved on 2025-07-09 from the Digital Agency Well-Being Dashboard (well-being.digital.go.jp). Results correspond to that snapshot; later updates/corrections may change values.
19	Sampling & recruitment	[Public design info] Do we report publicly available sample design details and identify items that are not disclosed?	[Digital Agency survey] Public design info includes: opt-in internet users (ages 18–89), quota-based recruitment, and year-specific design differences. Not disclosed/limited: selection probabilities and probability-sample response rates; no official representativeness statement.<br>[This study (outcome use)] We rely only on publicly documented design information and treat non-disclosed elements as an explicit limitation.
20	Outcome definition & aggregation	[Aggregation rule] Is the aggregation rule for the prefecture-level mean stated (mean; missing handling)?	Time window used: 2022–2025.<br>Computation: SWB_pref,year = mean(SWB_i | prefecture=pref, year=y, SWB_i not missing).<br>Aggregation pathway: direct aggregation from individual records to prefecture (not re-averaging municipality means).<br>Data retrieval date: 2025-07-09 (Digital Agency Well-Being Dashboard).
21	Limitations	[Structural bias/incentives] Do we acknowledge that panel operations and incentives may bias observed SWB levels?	[Digital Agency survey] Panel incentives and platform effects can shift who responds and how they respond.<br>[This study (outcome use)] Potential incentive-/panel-related bias is treated as a limitation; observed SWB levels may be systematically shifted upward/downward.
22	Provenance & ethics	[Terms of use/IP] Do we clearly state data provenance and comply with terms-of-use / IP constraints (SCI-Japan / Digital Agency dashboard, as applicable)?	[SCI-Japan disclaimer (summary)] The Regional Well-Being Indicator is a copyrighted work computed using an independently developed method by SCI-Japan; SCI-Japan holds all intellectual property rights to the indicator, related works, and the calculation method. When quoting, required attribution applies; uses beyond private use/quotation require permission. SCI-Japan does not guarantee accuracy/completeness and may change the method or suspend publication.<br>[This study (outcome use)] We use only publicly available aggregated prefecture means and provide the required source attribution; we do not redistribute underlying copyrighted materials beyond lawful quotation.
23	Comparability	[Year-to-year comparability] Do we justify (or appropriately limit) longitudinal comparability across years (2022–2025) despite possible design differences?	[Digital Agency reply] No official recommended approach is provided; users should interpret with caution given differences in target population and questionnaire specifications across years.<br>[This study (outcome use)] Year-to-year differences across 2022–2025 are treated as descriptive (not precise temporal trends or causal changes).

Table S5. Descriptive year-to-year comparability of published prefecture-level mean SWB (0–10), 2022–2025.

Panel A. Year×Year Pearson correlation (computed across 47 prefectures; coefficients

	**2022**	**2023**	**2024**	**2025**
2022	1.00000	0.53563	0.41731	0.25944
2023	0.53563	1.00000	0.77645	0.75459
2024	0.41731	0.77645	1.00000	0.76596
2025	0.25944	0.75459	0.76596	1.00000

Panel B. Cross-prefecture distribution summary by year (no prefecture identifiers)

**Year**	**Mean across prefectures**	**SD across prefectures**
2022	5.9236	0.1703
2023	6.4379	0.1035
2024	6.4611	0.1394
2025	6.3985	0.1177

Note: Pearson correlations and distribution summaries were computed across the 47 published prefecture-year mean SWB values (0–10) from the Digital Agency dashboard snapshot used in this study (see Table S4). No prefecture-level values, ranks, or league tables are shown. These descriptive statistics assess stability of released aggregates and do not constitute independent validation of the underlying survey measurement.

Table S6. Sensitivity analysis: selected raw Google Trends series versus PCA-based Trends features.

Training years: 2022–2024. Holdout evaluation year: 2025.

Panel A. Association of each Google Trends category series (SVI) with the first Trends principal component (PC1) (training years only).

svi_col	category_label	corr_with_pc1	abs_corr_with_pc1	rank_abs
svi_beauty_n_fitness	Beauty & Fitness	0.74603	0.74603	1
svi_automotive	Automotive	0.74231	0.74231	2
svi_food_n_drink	Food & Drink	0.74128	0.74128	3
svi_online_communities	Online Communities	0.69299	0.69299	4
svi_travel	Travel	0.68346	0.68346	5
svi_people_n_society	People & Society	0.60964	0.60964	6
svi_home_n_garden	Home & Garden	0.59310	0.59310	7
svi_business_n_industry	Business & Industry	0.57299	0.57299	8
svi_books_n_literature	Books & Literature	0.57086	0.57086	9
svi_science	Science	0.55689	0.55689	10
svi_pets_n_animals	Pets & Animals	0.54577	0.54577	11
svi_hobbies_n_leisure	Hobbies & Leisure	0.53423	0.53423	12
svi_arts_n_entertainment	Arts & Entertainment	0.52053	0.52053	13
svi_computers_n_electronics	Computers & Electronics	0.50976	0.50976	14
svi_law_n_government	Law & Government	-0.46167	0.46167	15
svi_real_estate	Real Estate	0.30332	0.30332	16
svi_games	Games	-0.23732	0.23732	17
svi_shopping	Shopping	0.21621	0.21621	18
svi_health	Health	-0.17479	0.17479	19
svi_jobs_n_education	Jobs & Education	0.15898	0.15898	20
svi_news	News	0.11571	0.11571	21
svi_internet_n_telecommunications	Internet & Telecommunications	-0.08643	0.08643	22
svi_finance	Finance	0.06232	0.06232	23
svi_reference_materials	Reference Materials	-0.03241	0.03241	24
svi_sports	Sports	0.02509	0.02509	25

Panel B. Holdout (2025) performance comparison: PCA-based Trends vs selected raw Trends series (n=8).

feature_set	model	n_features	holdout_r2	holdout_pw_mse
Stage2_FE+Year	ElasticNet	26	0.18899	0.01123
Stage3_TopKRaw	ElasticNet	34	0.19100	0.01120
Stage3_TrendsPCA	ElasticNet	82	0.25436	0.01032
Stage2_FE+Year	XGB	26	0.08872	0.01261
Stage3_TopKRaw	XGB	34	-0.10621	0.01531
Stage3_TrendsPCA	XGB	82	0.56699	0.00599

Panel C. Feature importance for the selected raw Trends series model (Stage 3; no PCA).

model	feature_set	feature	feature_label	importance_type	value	abs_value	is_trends_topk
ElasticNet	Stage3_TopKRaw	yr_sin_p2		coef	0.01622	0.01622	FALSE
ElasticNet	Stage3_TopKRaw	time_idx_c		coef	0.01431	0.01431	FALSE
ElasticNet	Stage3_TopKRaw	yr_cos_p4		coef	0.01431	0.01431	FALSE
ElasticNet	Stage3_TopKRaw	cpi_value		coef	0.01347	0.01347	FALSE
ElasticNet	Stage3_TopKRaw	ets_pred		coef	0.01171	0.01171	FALSE
ElasticNet	Stage3_TopKRaw	nikkei225		coef	0.01022	0.01022	FALSE
ElasticNet	Stage3_TopKRaw	yr_sin_p4		coef	-0.00899	0.00899	FALSE
ElasticNet	Stage3_TopKRaw	yr_cos_p2		coef	-0.00899	0.00899	FALSE
ElasticNet	Stage3_TopKRaw	graw_svi_home_n_garden	Home & Garden	coef	-0.00665	0.00665	TRUE
ElasticNet	Stage3_TopKRaw	region_Tohoku		coef	-0.00610	0.00610	FALSE
ElasticNet	Stage3_TopKRaw	graw_svi_people_n_society	People & Society	coef	-0.00484	0.00484	TRUE
ElasticNet	Stage3_TopKRaw	pref_emb_2		coef	0.00456	0.00456	FALSE
ElasticNet	Stage3_TopKRaw	pref_emb_6		coef	0.00420	0.00420	FALSE
ElasticNet	Stage3_TopKRaw	graw_svi_travel	Travel	coef	0.00358	0.00358	TRUE
ElasticNet	Stage3_TopKRaw	region_Kansai		coef	0.00318	0.00318	FALSE
ElasticNet	Stage3_TopKRaw	graw_svi_beauty_n_fitness	Beauty & Fitness	coef	-0.00286	0.00286	TRUE
ElasticNet	Stage3_TopKRaw	graw_svi_online_communities	Online Communities	coef	0.00265	0.00265	TRUE
ElasticNet	Stage3_TopKRaw	graw_svi_food_n_drink	Food & Drink	coef	0.00265	0.00265	TRUE
ElasticNet	Stage3_TopKRaw	pref_emb_1		coef	0.00257	0.00257	FALSE
ElasticNet	Stage3_TopKRaw	graw_svi_automotive	Automotive	coef	0.00233	0.00233	TRUE
ElasticNet	Stage3_TopKRaw	region_Kyushu		coef	0.00221	0.00221	FALSE
ElasticNet	Stage3_TopKRaw	pref_emb_4		coef	-0.00218	0.00218	FALSE
ElasticNet	Stage3_TopKRaw	labor_force_q1		coef	0.00181	0.00181	FALSE
ElasticNet	Stage3_TopKRaw	gpp_2021		coef	0.00150	0.00150	FALSE
ElasticNet	Stage3_TopKRaw	region_Chugoku		coef	0.00146	0.00146	FALSE
ElasticNet	Stage3_TopKRaw	labor_participation_rate_1q		coef	0.00125	0.00125	FALSE
ElasticNet	Stage3_TopKRaw	region_Hokkaido		coef	0.00117	0.00117	FALSE
ElasticNet	Stage3_TopKRaw	region_Shikoku		coef	0.00103	0.00103	FALSE
ElasticNet	Stage3_TopKRaw	graw_svi_business_n_industry	Business & Industry	coef	0.00090	0.00090	TRUE
ElasticNet	Stage3_TopKRaw	region_Kanto		coef	-0.00084	0.00084	FALSE
ElasticNet	Stage3_TopKRaw	pref_emb_3		coef	0.00056	0.00056	FALSE
ElasticNet	Stage3_TopKRaw	pref_emb_5		coef	-0.00047	0.00047	FALSE
ElasticNet	Stage3_TopKRaw	income_per_capita_kjpy_2021		coef	-0.00009	0.00009	FALSE
ElasticNet	Stage3_TopKRaw	labor_force_rate		coef	0.00000	0.00000	FALSE
XGB	Stage3_TopKRaw	cpi_value		gain	0.18843	0.18843	FALSE
XGB	Stage3_TopKRaw	region_Tohoku		gain	0.06134	0.06134	FALSE
XGB	Stage3_TopKRaw	ets_pred		gain	0.02090	0.02090	FALSE
XGB	Stage3_TopKRaw	region_Shikoku		gain	0.00782	0.00782	FALSE
XGB	Stage3_TopKRaw	region_Kanto		gain	0.00680	0.00680	FALSE
XGB	Stage3_TopKRaw	graw_svi_travel	Travel	gain	0.00472	0.00472	TRUE
XGB	Stage3_TopKRaw	pref_emb_6		gain	0.00402	0.00402	FALSE
XGB	Stage3_TopKRaw	pref_emb_2		gain	0.00376	0.00376	FALSE
XGB	Stage3_TopKRaw	income_per_capita_kjpy_2021		gain	0.00344	0.00344	FALSE
XGB	Stage3_TopKRaw	region_Kyushu		gain	0.00302	0.00302	FALSE
XGB	Stage3_TopKRaw	pref_emb_3		gain	0.00243	0.00243	FALSE
XGB	Stage3_TopKRaw	labor_force_rate		gain	0.00241	0.00241	FALSE
XGB	Stage3_TopKRaw	pref_emb_4		gain	0.00212	0.00212	FALSE
XGB	Stage3_TopKRaw	labor_force_q1		gain	0.00200	0.00200	FALSE
XGB	Stage3_TopKRaw	gpp_2021		gain	0.00193	0.00193	FALSE
XGB	Stage3_TopKRaw	graw_svi_people_n_society	People & Society	gain	0.00163	0.00163	TRUE
XGB	Stage3_TopKRaw	graw_svi_food_n_drink	Food & Drink	gain	0.00132	0.00132	TRUE
XGB	Stage3_TopKRaw	pref_emb_5		gain	0.00131	0.00131	FALSE
XGB	Stage3_TopKRaw	graw_svi_business_n_industry	Business & Industry	gain	0.00131	0.00131	TRUE
XGB	Stage3_TopKRaw	graw_svi_automotive	Automotive	gain	0.00124	0.00124	TRUE
XGB	Stage3_TopKRaw	region_Hokkaido		gain	0.00123	0.00123	FALSE
XGB	Stage3_TopKRaw	graw_svi_home_n_garden	Home & Garden	gain	0.00121	0.00121	TRUE
XGB	Stage3_TopKRaw	graw_svi_beauty_n_fitness	Beauty & Fitness	gain	0.00113	0.00113	TRUE
XGB	Stage3_TopKRaw	graw_svi_online_communities	Online Communities	gain	0.00110	0.00110	TRUE
XGB	Stage3_TopKRaw	yr_sin_p2		gain	0.00107	0.00107	FALSE
XGB	Stage3_TopKRaw	labor_participation_rate_1q		gain	0.00101	0.00101	FALSE
XGB	Stage3_TopKRaw	pref_emb_1		gain	0.00100	0.00100	FALSE
XGB	Stage3_TopKRaw	region_Chugoku		gain	0.00073	0.00073	FALSE
XGB	Stage3_TopKRaw	region_Kansai		gain	0.00031	0.00031	FALSE
XGB	Stage3_TopKRaw	nikkei225		gain	0.00000	0.00000	FALSE
XGB	Stage3_TopKRaw	time_idx_c		gain	0.00000	0.00000	FALSE
XGB	Stage3_TopKRaw	yr_cos_p2		gain	0.00000	0.00000	FALSE
XGB	Stage3_TopKRaw	yr_cos_p4		gain	0.00000	0.00000	FALSE
XGB	Stage3_TopKRaw	yr_sin_p4		gain	0.00000	0.00000	FALSE

Notes: PC1 was computed by PCA on the z-scored matrix of all 25 Google Trends category series (SVI) using training years only.

Selected raw series were the 8 series with the largest absolute correlation with PC1 (Panel A).

PW-MSE is mean squared error computed with equal weight per prefecture.

Abbreviations: PC1, first principal component; PCA, principal component analysis; SVI, Search Volume Index; PW-MSE, prefecture-weighted mean squared error; FE, fixed effects; SWB, subjective well-being.
