# Supplementary material for: Prediction of Prefecture-Level Subjective Well-Being in Japan by Using Google Trends and Socioeconomic Data: Machine Learning Model Development and Validation Study
Source: JMIR Form Res. 2026 Mar 20;10:e88696. doi: 10.2196/88696 (PMC13049395; doi:10.2196/88696)
Supplement: Multimedia Appendix 2 [file formative_v10i1e88696_app2.pdf]

Appendix Algorithm S1.
Walk-Forward Multi-stage Subjective Well-being Prediction Model

This algorithm describes the leakage-controlled walk-forward training and evaluation pipeline used to predict prefecture-level subjective well-being (SWB) in Japan from socioeconomic indicators and Google Trends data. It formalizes preprocessing, feature engineering, and stacked-ensemble integration.

Data source and units (TRIPOD-AI A1–A2):
- Publicly available aggregated panel data for all 47 prefectures of Japan over the study period (e.g., 2022–2025), obtained from governmental statistics portals and Digital Agency resources (URLs listed in the main text / Supplementary materials).
- The analysis unit is the prefecture (47 units). Inclusion, exclusion, and missing-data handling follow the preprocessing description in the main text.
- Subjective well-being outcomes are annual prefecture-level means from the Digital Agency surveys on a 0–10 scale.

Input:

```
    PREFECTURE_i         # Prefecture identifier (fixed-effect embedding + region dummies)
    ECO_it               # Socioeconomic indicators (e.g., CPI, income, labor statistics)
    TIME_it              # Calendar year (e.g., 2022–2025)
    TRENDS_it            # Google Trends panel: yyyy/mm × prefecture × search category
    SWB_it               # Mean SWB: yyyy × prefecture, 0–10 scale
```

Output:

SWB predictions per prefecture for each target year (2023, 2024, 2025)
Performance metrics (adjusted R^2 and MSE) at national and macro-regional levels, with uncertainty summaries Calibration statistics (slope, intercept) and calibration plots (predicted vs bserved) Incremental value summaries (Δ adjusted R^2 and Δ MSE) for Stages 2 and 3 relative to Stage 1
Regional error profiles for heterogeneity / fairness assessment

Notation (informal model view):

For prefecture i and year t,
let SWB_it denote the mean SWB outcome (0–10), and let ECO_it, PREFECTURE_i, TIME_it, and TRENDS_it denote the socioeconomic, fixed-effect (prefecture), temporal, and Google Trends predictor blocks, respectively, as defined in the main text.
We consider three nested feature sets:

```
      Stage 1: X_it^(1) = [PREFECTURE_i, ECO_it]
      Stage 2: X_it^(2) = [X_it^(1), TIME_it]
      Stage 3: X_it^(3) = [X_it^(2), TRENDS_it].
```

Base learners are fit on each feature set, and a stacked meta-learner estimates a data-driven combination of their predictions.

Procedure:

1. Load and preprocess input data:
    Cast ECO_it to numeric and apply configured transformations
    Construct PREFECTURE_i embeddings (learned vectors) and region one-hot encodings

2. Feature Engineering:
    Compute one-step-ahead ETS forecasts of SWB_it for TIME_it+1
    Derive SWB slope and volatility over a trailing window
    Create macro-region indicators (e.g., Kanto, Kansai)
    Apply PCA to normalized TRENDS_it and
    retain PCs explaining >90% variance (TRENDS_PCs_it)
    Define feature sets:
      FE = [PREFECTURE_i embedding, ECO_it variables]

```
      FE + TIME = FE + [ETS forecast, slope, volatility, year]
      FE + TIME + TRENDS = FE + TIME + [TRENDS_PCs_it, TRENDS_PCs_it × selected ECO_it]
    For each feature set (FE, FE + TIME, FE + TIME + TRENDS):
      Optionally run VIF diagnostics to flag collinear predictors (VIF > threshold)

3. Walk-Forward Validation and Base-learning:
    For each forecast year y ∈ {2023, 2024} (2025 as final holdout):
        Split data into a training fold (years t < y) and a validation fold (year t = y)
        Fit preprocessing components (e.g., scaling, ETS, PCA, PREFECTURE_i embeddings)
        using only the training fold to avoid temporal leakage
        Apply the fitted preprocessing to the validation fold
        Train 7 Optuna-tuned base learners on the training fold:
            ElasticNet, Ridge, SVR, XGBoost, Extra Trees, shallow LightGBM, deep LightGBM
        Obtain out-of-fold (OOF) predictions on the validation fold
        Compute evaluation metrics (adjusted R² and MSE) for each feature set, and
        summarize their uncertainty as defined in the main text

4. Meta-learning Integration:
    Use OOF predictions from the base learners as meta-features
    Cluster highly correlated base predictions and retain one representative per cluster
    Train and evaluate an ElasticNet meta-model
    with these features and PREFECTURE_i dummies

5. Final Evaluation:
    Apply preprocessing, base models, and the meta-model (ElasticNet)
    to the 2025 holdout data
    Compute final evaluation summaries:
        - SWB predictions per prefecture
        - Aggregated performance (adjusted R² and MSE) with uncertainty estimates
        - Calibration statistics (slope, intercept) and calibration
          plots comparing predicted vs observed SWB
        - Block-wise information gain is defined as
          ΔR²_B = R²_adj(baseline + B) − R²_adj(baseline), where B ∈ {Temporal, Trends}
        - Robustness checks via comparison of Stage 1→2→3 performance and inspection
          of the stability of TRENDS_PCs loadings across years
        - Regional error profiles (per prefecture and macro-region) used to discuss
          heterogeneity and fairness

6. Reporting, reproducibility, and intended use (TRIPOD-AI-oriented notes):
    - Make the full analysis repository, environment description, and end-to-end pipeline
      publicly available upon publication, as stated in the manuscript and Supplementary
      materials.

    - Explicitly document in the manuscript the scope and limitations of this model:
      aggregated (prefecture-level) SWB in Japan, the 2022–2025 study period, and the
      non-clinical policy / monitoring context.
    - Ensure that calibration, added value, robustness, and regional heterogeneity
      summaries produced above are reported in alignment with the TRIPOD-AI checklist.


End Algorithm
```
