# Supplementary figures and images for: Prediction of Prefecture-Level Subjective Well-Being in Japan by Using Google Trends and Socioeconomic Data: Machine Learning Model Development and Validation Study
Source: JMIR Form Res. 2026 Mar 20;10:e88696. doi: 10.2196/88696 (PMC13049395; doi:10.2196/88696)

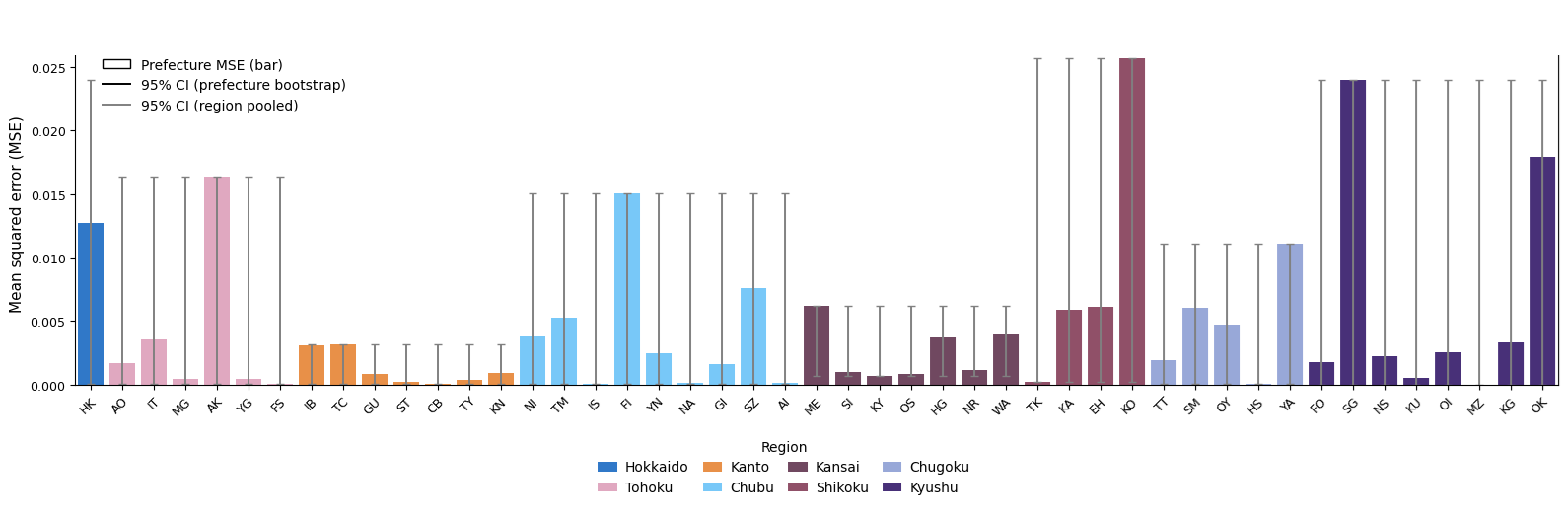

Supplement: Multimedia Appendix 4 [file formative_v10i1e88696_app4.png]
