# Supplementary material for: Prediction of Prefecture-Level Subjective Well-Being in Japan by Using Google Trends and Socioeconomic Data: Machine Learning Model Development and Validation Study
Source: JMIR Form Res. 2026 Mar 20;10:e88696. doi: 10.2196/88696 (PMC13049395; doi:10.2196/88696)
Supplement: Multimedia Appendix 5 [file formative_v10i1e88696_app5.pdf]

| Section | Item | Description |
|---|---|---|
| A. Study Design Transparency | 1. Data source & timeframe | Public aggregated data (Japan prefectures, 2022–2025) with direct URLs. |
| | 2. Participants/units | 47 prefectures; inclusion/exclusion and missingness handling. |
| | 3. Predictors | Socioeconomic indicators, temporal controls, PCA-based Google Trends. |
| | 4. Outcome | Subjective well-being (0–10), Digital Agency annual surveys. |
| B. Model Evaluation & Reproducibility | 5. Models | Seven base learners + Elastic Net meta-learner (stacking). |
| | 6. Data splitting | Strict walk-forward validation; 2025 final holdout. |
| | 7. Performance metrics | Adjusted $R^2$, MSE with uncertainty. |
| | 8. Calibration | Slope/intercept and calibration scatter assessment. |
| | 9. Incremental value | Stage 1→2→3 $\Delta R^2$ showing added value of Trends features. |
| | 10. Robustness | Nested feature-set comparisons (Stage 1→2→3) and stability checks for Google Trends PCA loadings. |
| | 11. Fairness/heterogeneity | Regional error spread and interpretability notes. |
| | 12. Reproducibility | Planned public release of the full analysis repository, environment file, and end-to-end pipeline upon publication. |
| | 13. Limitations & intended use | Scope, generalizability limits, and non-clinical context. |
